# FDA approval of lecanemab: the real start of widespread amyloid PET use? — the EANM Neuroimaging Committee perspective

**DOI:** 10.1007/s00259-023-06177-5

**Published:** 2023-03-04

**Authors:** Antoine Verger, Igor Yakushev, Nathalie L. Albert, Bart van Berckel, Matthias Brendel, Diego Cecchin, Pablo Aguiar Fernandez, Francesco Fraioli, Eric Guedj, Silvia Morbelli, Nelleke Tolboom, Tatjana Traub-Weidinger, Donatienne Van Weehaeghe, Henryk Barthel

**Affiliations:** 1 Department of Nuclear Medicine and Nancyclotep Imaging Platform, Université de Lorraine, CHRU; IADI, INSERM U1254, Nancy, France; 2 Department of Nuclear Medicine, School of Medicine, Technical University of Munich, Ismaninger Str. 22, 81675 Munich, Germany; 3 Department of Nuclear Medicine, Ludwig Maximilians-University of Munich, Munich, Germany; 4 Department of Radiology and Nuclear Medicine, University Medical Center Utrecht, Utrecht, the Netherlands; 5 Nuclear Medicine Unit, Department of Medicine - DIMED, University of Padua, Padua, Italy; 6 Department of Radiology, Faculty of Medicine and Center for Research in Molecular Medicine and Chronic Diseases (CIMUS), University of Santiago de Compostela (USC), Campus Vida, Santiago de Compostela, Galicia Spain; 7 Institute of Nuclear Medicine, University College London (UCL), London, UK; 8 APHM, CNRS, Centrale Marseille, Nuclear Medicine Department, Institut Fresnel, Timone Hospital, CERIMED, Aix Marseille Univ, Marseille, France; 9 Nuclear Medicine Unit, Department of Health Sciences, University of Genoa, Genoa, Italy; 10 Nuclear Medicine and Molecular Imaging, University Medical Center Groningen, Groningen, the Netherlands; 11 Division of Nuclear Medicine, Department of Biomedical Imaging and Image-Guided Therapy, Medical University of Vienna, Vienna, Austria; 12 Department of Radiology and Nuclear Medicine, Ghent University Hospital, Ghent, Belgium; 13 Department of Nuclear Medicine, Leipzig University, Leipzig, Germany

Lecanemab is a humanized IgG1 monoclonal antibody that targets protofibrils, a species of soluble aggregated amyloid-beta (Aβ). The drug was approved by the U.S. Food and Drug Administration (FDA) for the treatment of Alzheimer’s disease (AD) on January 6, 2023. This approval was based on results of a phase 2 clinical trial [1] and followed the publication of results of a phase 3 trial in November 2022 [2]. The latter study included 1795 patients with early AD, i.e., mild cognitive impairment and mild dementia due to AD. This was the first Aβ immunotherapy study to report significant slowing of progression on the clinical dementia rating scale-sum of boxes at 18 months in the whole cohort, with a mean change from baseline between patients treated with lecanemab vs. placebo of − 0.45 (*p* < 0.001) [2]. Moreover, in a sub-cohort of 698 patients, reduction in amyloid burden was detected by PET in the lecanemab arm but not in the placebo arm. An extension study of the long-term efficacy, safety, and tolerability of lecanemab is ongoing, with a sub-study of a subcutaneous administration of lecanemab being monitored exclusively by amyloid PET.

Lecanemab is the second FDA-approved anti-Aβ drug, after aducanumab [3]. Like lecanemab, aducanumab was shown to reduce amyloid burden on PET. However, evidence of a clinical benefit of aducanumab was mixed, with a significant slowing of cognitive decline only being shown in one of two identically designed phase 3 clinical trials [3]. Aducanumab has therefore been not indicated for clinical use in the USA and not approved in Europe. A phase 3b/4 study (ENVISION) is ongoing to verify the clinical benefit of aducanumab in early AD. Different from aducanumab, the evidence for efficiency of lecanemab has been consistent across studies and outcomes [1, 2, 4]. The beneficial effect of lecanemab has been associated with its binding profile. Specifically, lecanemab mainly targets Aβ protofibrils, while aducanumab and other monoclonal antibodies favour highly aggregated forms of Aβ [5]. This differing target profile may also explain a substantially lower incidence of amyloid related imaging abnormalities, such as transient immunotherapy-related brain oedema and microbleeds, with lecanemab [5].

A number of phase 3 clinical trials of anti-Aβ therapies in early AD have been performed or are ongoing. All have used amyloid PET as an inclusion criterion and a secondary endpoint, at least in a subgroup of patients [6–10]. Also pending are results of the phase 3 trial of donanemab, an alternative anti-Aβ therapy, for which post hoc analyses of the phase 2 trial found associations between greater amyloid plaque clearance on amyloid PET and slower progression of tau PET as well as slower clinical decline in apolipoprotein E ε4 carriers [11]. Overall, there is increasing evidence that the anti-Aβ therapies slow down cognitive decline in early AD [12].

What are the implications of the FDA approval of lecanemab for the use of amyloid PET? Lecanemab is currently under review by the European Medicines Agency (EMA). If approved, two scenarios of possible reimbursement of amyloid PET in Europe may be envisaged, either as a part of an anti-Aβ therapy, i.e., within an all-inclusive service package, or as a dedicated diagnostic test. In any case, the introduction of amyloid PET–guided lecanemab treatment will be a challenge for health systems. The Nuclear Medicine community should therefore be prepared to adjust its capacities to the increased demand for amyloid PET radiotracers, PET imaging infrastructure, and training for image reading in the near future. According to some estimates, the need for amyloid PET may increase by a factor of 20 [13]. Even though lumbar puncture (LP) for cerebro-spinal fluid (CSF) amyloid proteins is the primary tool to diagnose the Aβ pathology, up to one-third of patients may require amyloid PET [14]. These are individuals who refuse LP, patients with contraindications against LP or with inconclusive CSF results.

It remains to be seen whether the indication for amyloid PET will be extended to individualized therapy monitoring in the future. More research is certainly required in this field, but we see a great potential for repeated amyloid PET (a) to identify potential non-responders with consecutive interruption of the therapy, (b) to guide the dosage and duration of the therapy, or (c) to plan a resumption of the therapy after a temporary therapy response. In this case, the Nuclear Medicine community would need to adopt a consensus in regard to quantitative amyloid PET analysis to correctly track longitudinal changes in a scenario where changes in blood flow are expected [15]. Furthermore, it is worth noting that the currently approved tracers predominantly detect amyloid PET changes induced by fibrillary plaque components, whereas toxic oligomers only weakly contribute to the overall amyloid PET signal [16].

Over the past 5 years, international efforts by Nuclear Medicine scientists and physicians have promoted amyloid PET by means of expert consensus [14] and real-life/prognostic impact studies [17–20]. The EANM Neuroimaging Committee believes that amyloid PET is now at a historical turning point, where it has paved the way for early AD diagnosis and now may also have also therapeutic implications. We expect that amyloid PET will be more widely used in the near future to justify the initiation and to monitor the effect of disease-modifying therapies on biological grounds. If the EMA approves lecanemab following the FDA, European authorities are likely to expand access to amyloid PET, overcoming the current restrictions in numerous countries.
